# Transition Metal Catalyzed Synthesis of Aryl Sulfides

**DOI:** 10.3390/molecules16010590

**Published:** 2011-01-17

**Authors:** Chad C. Eichman, James P. Stambuli

**Affiliations:** Department of Chemistry, The Ohio State University, 100 West 18th Avenue, Columbus, OH 43210, USA

**Keywords:** C-S bond formation, biaryl sulfides, arylation of thiols

## Abstract

The presence of aryl sulfides in biologically active compounds has resulted in the development of new methods to form carbon-sulfur bonds. The synthesis of aryl sulfides via metal catalysis has significantly increased in recent years. Historically, thiolates and sulfides have been thought to plague catalyst activity in the presence of transition metals. Indeed, strong coordination of thiolates and thioethers to transition metals can often hinder catalytic activity; however, various catalysts are able to withstand catalyst deactivation and form aryl carbon-sulfur bonds in high-yielding transformations. This review discusses the metal-catalyzed arylation of thiols and the use of disulfides as metal-thiolate precursors for the formation of C-S bonds.

## 1. Introduction

**Figure 1:**
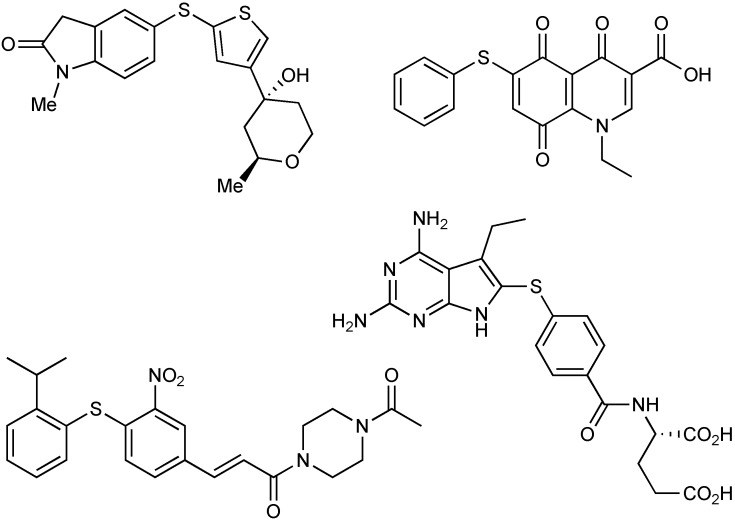
Aryl sulfide-containing pharmaceuticals.

Over the last thirty years, significant strides have been made in organometallic processes that form carbon-sulfur bonds. Substantial growth in the transition metal-catalyzed formation of carbon-heteroatom bonds has been observed, however, the development of effective C-S bond formation reactions is underdeveloped with respect to the corresponding C-N and C-O coupling reactions. The necessity for the advancement of carbon-sulfur bond forming reactions is warranted by the prevalence of biarylsulfides in natural and unnatural products that exhibit activities against cancer, HIV, Alzheimer’s disease, inflammation, and asthma [1,2,3,4,5,6,7,8,9,10,11]. Figure 1 represents some biologically active sulfide-containing compounds. 

Methods to synthesize aryl sulfides without the use of transition metals are generally inefficient, require impractical reaction conditions, and have limited functional group tolerance. Some of these methods include nucleophilic attack on disulfides, aromatic substitution reactions, and metal-mediated disulfide reductions. The development of practical and efficient methods to create aryl sulfides has been realized through transition metal catalysis. 

Transition metal-thiolate interactions are strong and numerous stable complexes have been reported in the literature. This strong coordinating ability often leads to the belief that sulfur will hinder transition metal catalytic activity. Despite this notion, thioethers can be excellent ligands for metal-catalyzed processes and metal-thiolate complexes can undergo facile reductive elimination to form C-S bonds. 

Reviews discussing metal-catalyzed carbon-heteroatom bond forming reactions have been reported, including a recent excellent review on organometallic approaches to C-S bond formation [12]. This review is meant to serve as an account to discuss the proposed mechanistic aspects that allow arylsulfide formation through transition metal catalysis. The significant advances in the field will be described and when possible, mechanistic rationale will be discussed for each C-S bond forming process presented. 

## 2. Palladium-Catalyzed Arylation of Thiols

**Scheme 1:**
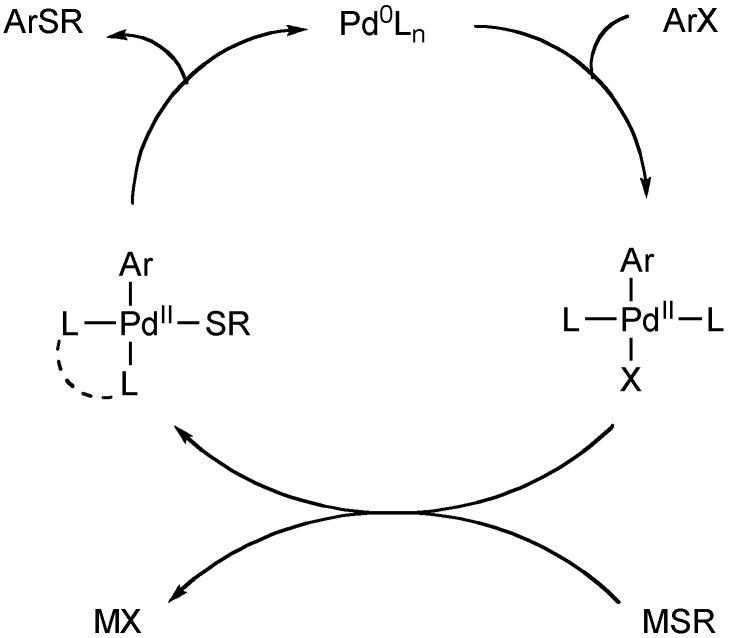
General mechanism for Pd-catalyzed arylthioether synthesis.

In 1978, Migita reported the palladium-catalyzed thiation of aryl halides using Pd[PPh_3_]_4_ as a catalyst [13,14]. The method provides biaryl sulfides in good yields, but is limited to aryl bromides and also requires high reaction temperatures and long reaction times (Equation 1). Typically, the palladium-catalyzed methods following Migita’s report utilize catalysts containing bidentate phosphine ligands [15,16,17,18,19,20,21,22,23,24,25,26,27,28,29,30,31,32,33,34,35,36,37,38,39,40,41,42,43,44,45,46,47,48,49,50,51,52,53]. The bidentate phosphine-ligated systems are proposed to be successful because of their ability to stay coordinated to the metal upon attack of the thiolates at palladium. These reactions are thought to proceed through the standard mechanism for typical palladium-catalyzed carbon-heteroatom bond formations (Scheme 1). 


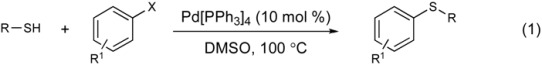


Buchwald disclosed the first practical aryl sulfide synthesis from aryl chlorides [34]. This report tested a variety of monodentate and bidentate phosphine ligands, with the bidentate DiPPF ligand providing the optimal catalyst system (Equation 2). The transformation is highly efficient and functional group tolerant. The synthesis of biarylsulfides from electron rich aryl chlorides required a weaker base (*^n^*Bu_3_N), higher temperatures and longer reaction times to provide high yields. It is noteworthy that all bulky monodentate phosphine ligands formed unreactive catalysts. This observation was rationalized that the highly nucleophilic thiolate anions displaced the monodentate ligands and created an inactive palladium species.


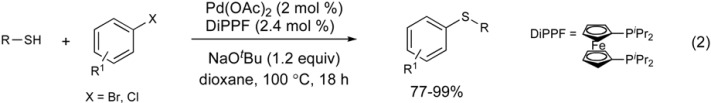


The most significant advance in the palladium-catalyzed C-S bond formation was discovered by Hartwig and co-workers in 2006 [54,55]. Employing the strongly coordinating bidentate Josiphos ligand in the presence of a palladium salt created a highly stable and reactive catalyst. The reaction proceeds at extremely low catalyst loadings down to parts per million in palladium and can couple aryl chlorides with thiols in excellent yields (Equation 3). Functional group tolerance is very good as the reaction is effective in the presence of unprotected phenols, carboxylic acids, anilines, and amides. The rationale for the high reactivity of the catalyst system is attributed to the strong coordination ability of the Josiphos ligand. 

**Scheme 2:**
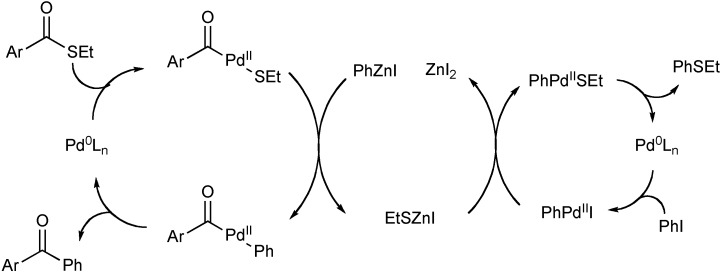
Potential pathway for the formation of aryl sulfides in the Fukuyama reaction.

In our recent work, the authors’ discovered that aryl sulfides were formed as a byproduct of a Fukuyama coupling reaction [52]. During the course of the Fukuyama coupling [56], palladium activation of a thioester and transmetalation with an organozinc reagent produced a zinc thiolate species as a byproduct (Scheme 2).


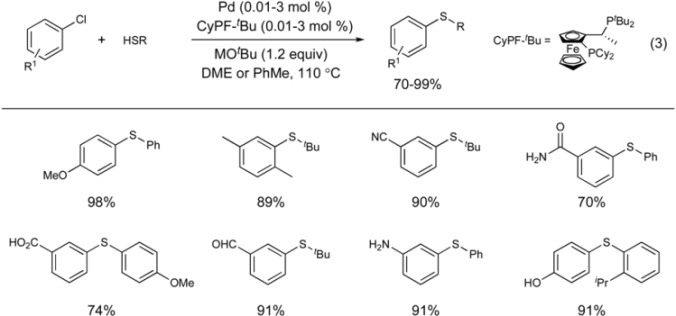


The zinc thiolate was found to act as a less nucleophilic sulfur anion in a C-S bond forming process. More importantly, the arylation of the zinc thiolate proceeded in the presence of tri-*tert*-butyl phosphine as a ligand. This observation led to a series of experiments to determine the factors that allow facile C-S bond formation to occur in the presence of a monodentate phosphine. It was discovered that a substoichiometric amount of zinc chloride alleviates strong coordination from thiolates on palladium and prevents catalyst deactivation. This method represents a rare example of a general, palladium-catalyzed aryl sulfide synthesis using a monodentate phosphine ligand.

Lautens and co-workers described an intramolecular C-S bond forming process with monodentate SPhos as a ligand in the palladium mediated coupling [50]. This tandem aryl sulfide formation/Suzuki-Miyaura protocol is an excellent way to create functionalized benzothiophenes in high yields (Equation 4). Similar ligands to SPhos were shown to be unreactive in palladium-catalyzed processes, however, a fast intramolecular C-S bond forming process presumably allows the transformation to occur. 


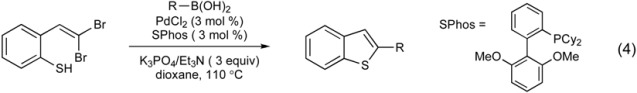


Lin and co-workers recently reported the first Pd/C catalyzed arylation of thiols [53]. This ligand-free process is limited to the use of aryl iodides and activated aryl bromides. 

Hartwig and co-workers performed the first detailed mechanistic investigation for palladium-mediated C-S bond formation [18,20,23]. These initial studies used tin thiolate species to investigate the transmetalation and reductive elimination steps for the process. The reductive elimination step was examined by observing the rate of arylsulfide formation from isolated Pd(II) thiolato aryl complexes (Equation 5). This pioneering mechanistic analysis of carbon-heteroatom bond forming processes revealed a significant difference between the reductive elimination of these bonds compared to C-C and C-H bonds. It was evident that the rate acceleration of the reductive elimination of electron-rich thiolates on electron poor carbons indicates nucleophilic attack by the thiolate on the carbon during reductive elimination.


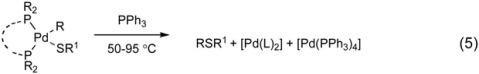


**Scheme 3:**
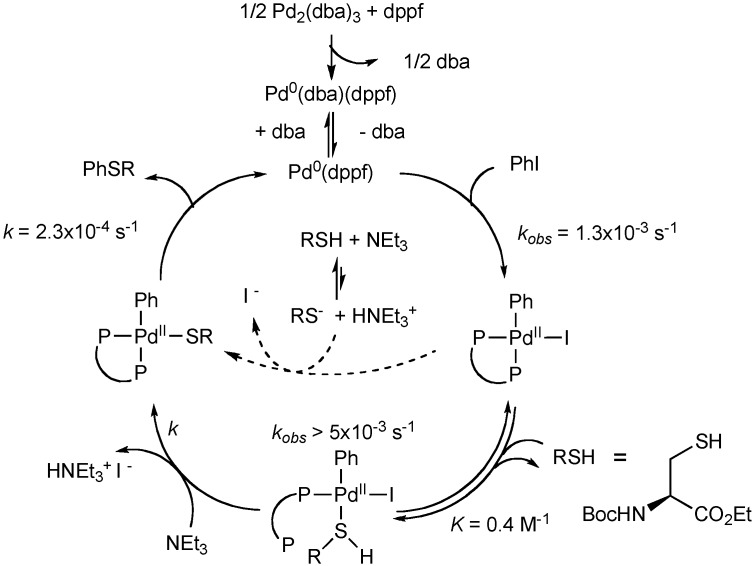
Mechanism of the palladium-catalyzed arylation of cysteine.

Campagne and Jutand reported mechanistic studies on the palladium-mediated C-S bond forming reaction using a cysteine-derived thiol [37]. Palladium complexes of each step in the proposed catalytic cycle were detected through NMR spectroscopy and electrochemical techniques and the relative rates of reaction were measured. Interestingly, they report that a palladium thiol complex forms before deprotonation to generate a thiolate. The thiol-bound palladium complex [PhPdI(SHR)(η^1^-dppf)] was observed by ^31^P-NMR spectroscopy and it rapidly forms PhPd(SR)dppf upon the addition of Et_3_N. This reversible metallation of the thiol is proposed to facilitate the deprotonation step and would thus be applicable when weakly acidic thiols are employed. Further, the rate of reductive elimination to form the aryl sulfide was determined to be the slow step of the process. The final proposed catalytic cycle is depicted in Scheme 3.

More recently, Hartwig has performed intensive mechanistic studies of the Josiphos-ligated catalyst system [49]. The Josiphos ligand (CyPF-*^t^*Bu) is an electon-rich alkylbisphosphine that creates a highly reactive palladium complex for the arylation of thiols. Through the isolation and reaction of each palladium complex of the catalytic cycle, it was observed that each step (oxidative addition, transmetalation, reductive elimination) proceeds within minutes at or below ambient temperature. However, the overall catalytic reaction requires temperatures of 110 °C. Based on these results, it is clear that the resting state of the reaction lies off the catalytic cycle. The resting state of the catalytic process was probed through analysis of the rate of reaction of stable complexes that preceed the catalytic cycle. Through a series of studies from isolated palladium complexes it was determined that the resting state of the reaction depended greatly on the source of palladium. A palladium-dithiolate complex represents the resting state of reactions using Pd(OAc)_2_ as the source of palladium. For reactions using Pd(dba)_2_ as the Pd-source, (LPd)_2_(dba) was determined to be the resting state. Lastly, for reactions catalyzed by the complex of the initial oxidative addition of aryl halide [Pd(L)(X)(Ar)], the resting state lies at a palladium hydridothiolate complex that arises from oxidative addition of Pd(0) to the S-H thiol bond. Furthermore, these results strongly correlate to the catalytic reactions of other electron-rich bisphosphines. The catalyst system developed by Buchwald using DiPPF was demonstrated to act similarly to the CyPF-*^t^*Bu-ligated catalyst. The classical approach to improving a catalyst by accelerating the slow step of the catalytic cycle would fail for these reactions. For this system, acceleration of the rate of stable Pd-complexes to enter the catalytic cycle is necessary.

**Scheme 4:**
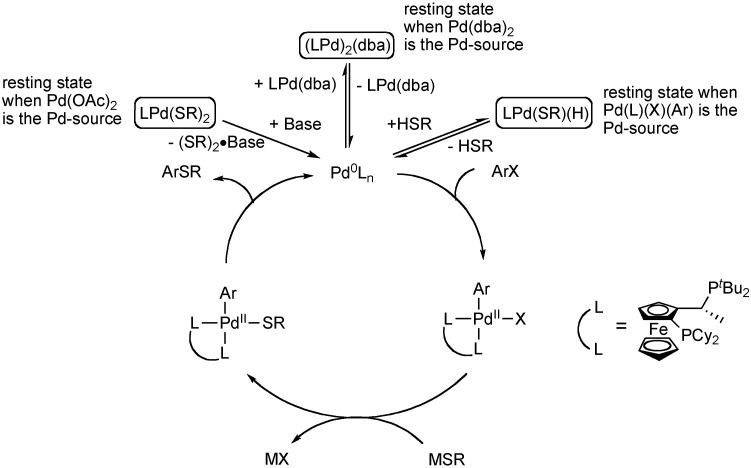
Mechanistic details for Josiphos-ligated Pd-catalyzed C-S bond formation.

## 4. Nickel-Catalyzed Arylation of Thiols

Shortly following Migita’s report on palladium-catalyzed arylation of thiols, Cristau and co-workers reported a nickel-catalyzed process to synthesize biaryl sulfides [57]. Using a nickel(II) complex with a bidentate phosphine ligand, arylsulfide formation is possible at 0.3 mol % catalyst (Equation 6). The reaction requires high temperatures and long reaction times and the yields are good to excellent.


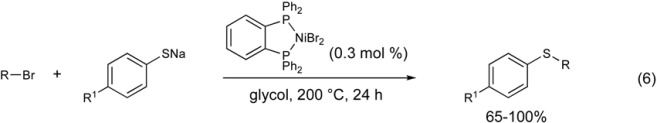


**Scheme 5:**
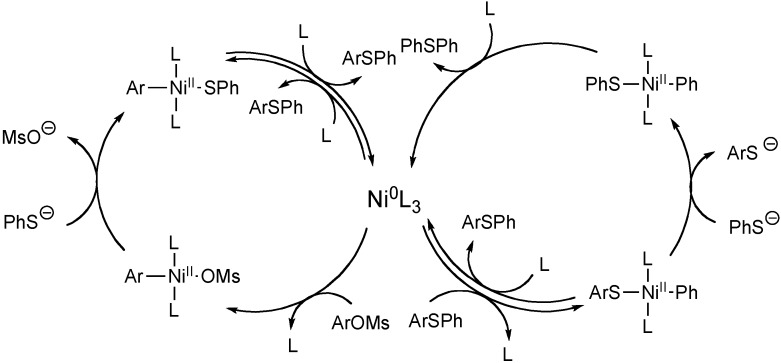
Mechanism of C-S bond formation using aryl mesylates.

Percec reported that aryl mesylates are feasible coupling partners for nickel catalyzed C-S bond forming reactions [58]. Using 10 mol % diphenylphospinoferrocenyl nickel(II) chloride in combination with 20 mol % dppf and 1 equiv zinc metal, sodium benzenethiolate was reacted with phenylmethane sulfonate to generate diphenylsulfide in 94% yield (Equation 7). Interestingly, other aryl mesylates also produced appreciable quantities of diphenylsulfides (Equation 8). This observation was rationalized through a mechanism suggesting that C-S bond activation occurs, followed by thiolate displacement and subsequent reductive elimination of diphenylsulfide (Scheme 5). Because of this mode of reactivity, the substrate scope for this reaction is limited. 


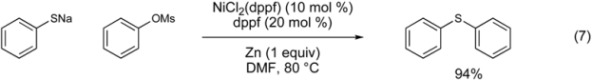



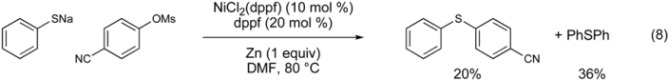


Most nickel-catalyzed protocols require high catalyst loadings and long reaction times [59,60,61,62,63,64,65]. Recently, it was shown that strongly coordinating NHC-ligands can allow a more efficient reaction to occur (Equation 9) [66]. 


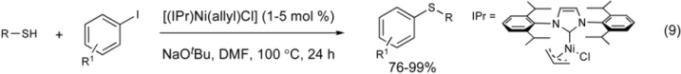


**Scheme 6:**
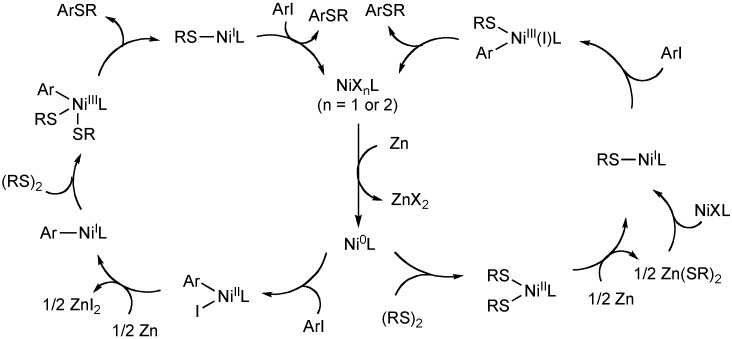
Catalytic cycle for nickel-catalyzed aryl thioether synthesis using disulfides.

Nickel catalysts have also been shown to insert into disulfide bonds and undergo C-S bond formation. Recently, Taniguchi has reported the arylation of disulfides in the presence of bipyridyl nickel(II) bromide (Equation 10). Various aryl iodides are transformed into their corresponding aryl sulfide using 0.5 equivalents of alkyl or aryl disulfides. The reaction is postulated to proceed through an initial zinc mediated reduction of nickel(II) to the active nickel(0) complex (Scheme 6). At this stage, oxidative insertion into the aryl iodide or the disulfide is possible. In both cases, another reduction of nickel(II) is proposed and a nickel(I) species can then undergo oxidative addition of disulfide or aryl iodide to produce a nickel(III) complex. Reductive elimination then provides the aryl sulfide.


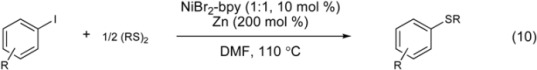


Nickel pincer complexes have also been shown to catalyze C-S bond forming reactions using disulfides and aryl iodides Equation (11) [62]. 


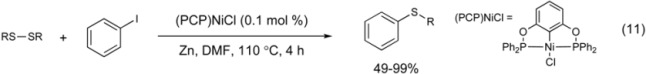


## 4. Copper-Catalyzed Arylation of Thiols

Over the last decade, copper has emerged as a viable catalyst for the arylation of thiols. [27,67,68,69,70,71,72,73,74,75,76,77,78,79,80,81,82,83,84,85,86,87,88,89,90,91,92,93,94,95,96,97,98,99,100,101,102,103] Palomo and co-workers demonstrated the ability of CuBr with phosphazene base to catalyze reaction between aryl iodides and thiols to afford biaryl sulfides Equation (12) [67]. Activated aryl bromides were also effective as coupling partners. Despite the high cost of the base and high catalyst loading, the reaction is efficient and established the basis for copper-catalyzed C-S bond formation.


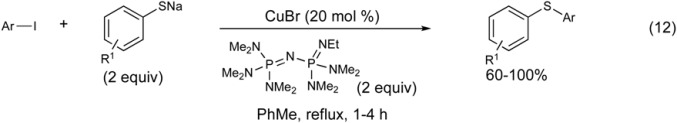


Buchwald reported the first practical synthesis of aryl sulfides using a copper catalyst (Equation 13) [68]. Under this protocol, 5 mol % CuI with two equivalents of ethylene glycol and K_2_CO_3_ can couple thiols with aryl iodides in good to excellent yields. The substrate scope is excellent with good functional group tolerance. Notably, the reaction proceeds in the presence of anilines and phenols. The ethylene glycol likely acts as a ligand to stabilize copper during the course of the reaction.


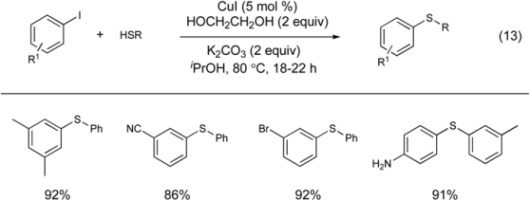


Recently, a highly regioselective process for the thiation of aryl halides was reported by Ranu and co-workers (Equation 14) [104]. Under this protocol, simply employing a different base significantly changes the reactivity of the copper catalyst. This alumina-supported copper catalyst has previously been employed in amination and etherification reactions. For thiation reactions, the use of K_2_CO_3_ allows the coupling of iodoarenes with thiols to occur in the presense of aryl bromides. Switching the base to Cs_2_CO_3_ under the same conditions gives a chemoselective coupling of the aryl bromide with aryl thiols. The chemoselectivity is attributed to the ability of a stronger base (Cs_2_CO_3_) to polarize the aryl bromide bond and allow copper to undergo a more facile oxidative addition of the aryl bromide compared to the K_2_CO_3_ system. Notably, the aryl amine is also not coupled with aryl bromide under these conditions.


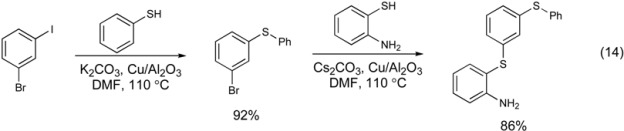


Disulfides and thioimides have been employed as thiolate surrogates in the copper-catalyzed thiation of arylboronic acids. This modified Chan-Evans-Lam cross-coupling reaction demonstrates the diverse utility of copper salts to construct carbon-heteroatom bonds. Guy first demonstrated the ability of arylboronic acids to react with thiols to construct the arylsulfide bond in the presence of stoichiometric copper. Based on this report, Liebeskind utilized thioimides as a copper-thiolate precursor (Equation 15). [105] A copper(I)-carboxylate complex catalyzes the cross-coupling of aryl boronic acids with thioimides to generate biaryl sulfides with moderate efficacy.


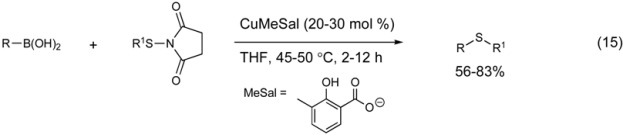


**Scheme 7:**
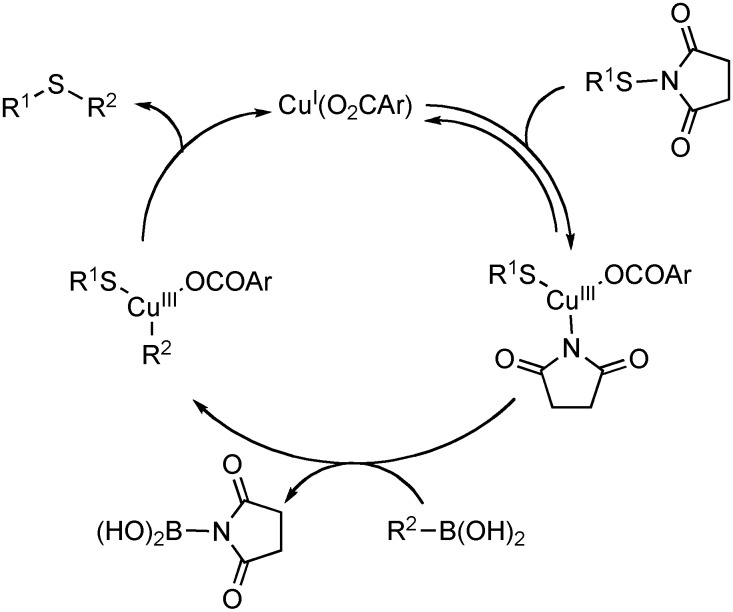
Catalytic cycle for the coupling of thioimides with boronic acids.

Mechanistically, the reaction is presumed to proceed through a Cu(I)-Cu(III) catalytic cycle (Scheme 7). Initial oxidative addition of the S-N bond of the thioimide, followed by transmetalation with the boronic acid generates the arylcopper(III) thiolate complex. Reductive elimination forms the aryl C-S bond and regenerates the active Cu(I)-catalyst. Consistent with this proposal, other copper carboxylates were effective in synthesizing aryl sulfide bonds. 

## 5. Miscellaneous Transition Metal Catalysts

### 5.1. The case of iron vs. copper

Bolm reported the use of catalytic iron(III) chloride in the *S*-arylation of thiols (Equation 16) [106]. The reaction was only compatible with aryl iodides and aryl thiols to construct biaryl sulfides.


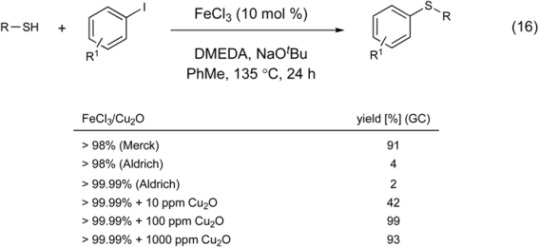


Shortly after this report, experiments performed in the Buchwald laboratory determined that copper, as little as 10 parts per million, was essential for catalytic activity [107]. Although the presence of copper may play a role in the iron-catalyze process, the efficacy of a C-S bond formation requiring only 10 mol % FeCl_3_ makes for an attractive, cost-friendly process. 

### 5.2. Cobalt-catalyzed aryltion of thiols

Cheng and co-workers disclosed a cobalt catalyzed process for the arylation of thiols (Equation 17) [108]. This method is successful for the coupling of aryl and alkyl thiols with aryl iodides and bromides. 


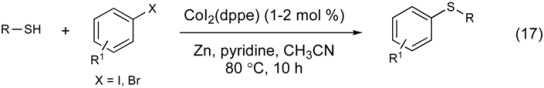


**Scheme 8:**
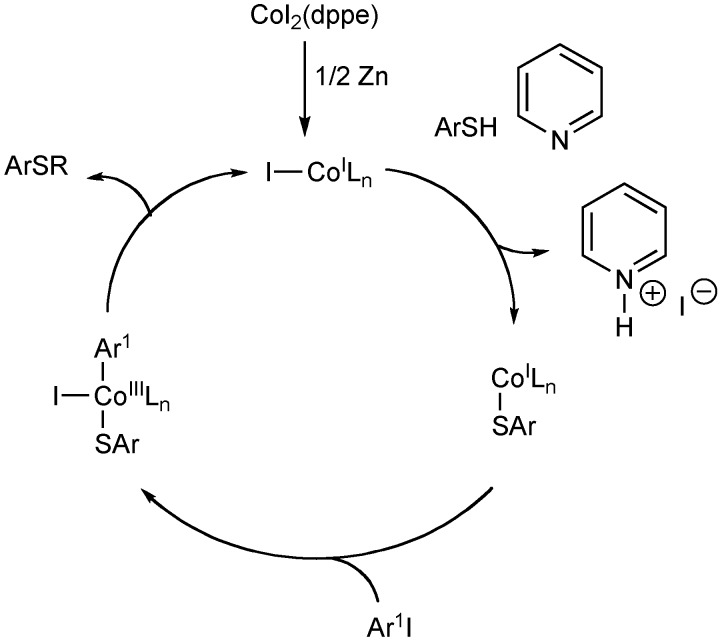
Cobalt-catalyze arylation of thiols

The mechanism of the cobalt catalyzed reaction is thought to occur through a cobalt(I)-(III) catalytic cycle (Scheme 8). Zinc metal reduces the starting cobalt(II) complex to the active cobalt(I) species. The reaction is then believed to undergo thiolate attack followed by oxidative addition of the aryl iodide. Reductive elimination affords the arylsulfide to complete the catalytic cycle.

## Conclusions

Extensive work in metal-catalyzed C-S bond forming reactions has resulted in significant advances for late-metal catalyzed processes. It is evident that the palladium-catalyzed reaction creates systems with the highest yields, lowest catalyst loadings, and highest functional group tolerance. Despite this fact, expensive chiral ligands are required for high activity. Progress has been made to eliminate the requirement of these ligands through the use of zinc chloride and through the development of “ligand-free” catalysts. Other metals are growing in synthetic utility for C-S bond forming processes, such as copper and nickel. Mechanistic investigations are becoming more common in order to fully understand these catalytic processes and to ultimately advance the development of more efficient catalysts.
